# The psychometric properties of the child anxiety and depression life interference scale (CADLIS)

**DOI:** 10.1186/s13034-024-00856-3

**Published:** 2025-01-29

**Authors:** Maddison O’Gradey-Lee, Alana Jones, Esther Gandhi, Heidi Lyneham, Ronald M. Rapee, Jennifer L. Hudson

**Affiliations:** 1 Black Dog Institute, University of New South Wales, Sydney, NSW 2031 Australia; 2 Centre for Emotional Health, Department of Psychology, Macquarie University, New South Wales, Macquarie Park, 2109 Australia; 3 School of Psychology, Faculty of Science, University of New South Wales, Sydney, NSW 2031 Australia

## Abstract

**Objective:**

Life interference is a key diagnostic feature for anxiety and depressive disorders. Measures focusing on life interference caused by anxiety and depressive disorders in children and adolescents have received minimal attention. This study evaluated the psychometric properties of the Child Anxiety and Depression Life Interference Scale (CADLIS), a brief child (CADLIS-C) and parent-report (CADLIS-P) measure designed to assess life interference from anxiety and depressive disorders in both the child and parent’s life.

**Method:**

A total of 672 parents of children aged 4–18 years completed the CADLIS-P, and 627 children aged 7–18 years completed the CADLIS-C.

**Results:**

The proposed two-factor CADLIS-C model was not supported, instead, due to high inter-factor covariance a one-factor model of life interference was proposed. The one-factor model demonstrated better model fit. The proposed three-factor model for the CADLIS-P was not supported, instead, an exploratory factor analysis found a two-factor model differentiating life interference into child and parent life interference a better model fit. The CADLIS demonstrated excellent internal consistency, good convergent and divergent validity, interrater correlations and was able to differentiate between children with and without clinical levels of anxiety and depressive symptoms.

**Limitations:**

Limitations of the study included the sample population which consisted of a small clinical sample, an over-representation of high-income families and the use of a panel provider.

**Conclusions:**

Overall, the CADLIS demonstrated sound psychometric properties. The CADLIS is a reliable measure that demonstrates evidence of convergent validity for the assessment of life interference associated with anxiety and depressive symptoms in children.

Anxiety and depressive disorders constitute some of the most common psychological disorders in children and young people [1–4]. Research indicates that depressive disorders are highly comorbid with anxiety disorders, particularly for adolescents [5, 6]. Symptoms associated with anxiety and depressive disorders can cause significant life interference also known as impairment for children and their parents in various contexts such as their social, academic and family functioning [7]. Life interference is the degree to which symptoms interfere with daily life [8]. Interference is an essential diagnostic criterion within the Diagnostic and Statistical Manual of Mental Disorders [9] and is used to differentiate between normative childhood worries, low mood and irritability and clinically significant anxiety and depressive disorders [10, 11]. Reductions in life interference are associated with a stronger sense of treatment satisfaction as life interference is often a key motivator to seek treatment [12, 13].

Individually both anxiety and depressive disorders cause considerable life interference, but when combined, the life interference experienced is more significant than the interference from a single disorder [11, 14]. In children with anxiety and depressive disorders high rates of school absenteeism, low rates of participation in extracurricular activities, poorer school performance, poorer family functioning and more significant peer problems [15–18].

The significant life interference from childhood anxiety and depressive disorders has led to a strong focus on the early identification, assessment and treatment of these disorders, resulting in numerous psychometrically sound assessment measures of symptoms that capture both the parent and child's perspectives [19]. However, life interference resulting from childhood disorders has received less attention, as there are no validated measures to assess the life interference of comorbid child anxiety and depressive disorders [13]. Creswell et al. [20] recommends that within the assessment of child anxiety disorders, a measure of life interference should be used. This is because interference measures, as opposed to symptom measures, align better with diagnostic criteria and outcomes such as treatment satisfaction [21, 22].

Pre-existing measures of life interference include The Child Sheehan Disability Scale (CSDS) [8], the Child Anxiety Impact Scale– Parent Version (CAIS-P) [23], Adolescent Life Interference Scale for Internalizing Symptom (ALIS-I) [24] and The Child Anxiety Life Interference Scale (CALIS) [13]. Whilst the CSDS, ALIS-I and the CAIS-P have many strengths, there are limitations concerning their assessment of life interference. These include i) the use of a global measure rather than a specific life interference measure; ii) the combination of symptomatology, distress, severity and interference, iii) failure to capture reports from multiple informants and iv) the sole focus on anxiety disorders v) the ALIS-I is designed for adolescents not children.

The CALIS [13] measures life interference related to anxiety disorders in children using both parent and child self-report measures. It is a brief measure that assesses interference in a child’s life and their parents’ life in various domains, such as in home and out of home interference as well as interference in the parent’s life. It also includes an item on distress. The CALIS is a reliable and valid tool for assessing life interference as it has both adequate reliability and moderate-to-high test–retest and inter-rater reliability, and is sensitive to treatment change [13]. A working group established by the International Consortium of Health Outcome Measures (ICHOM) determined the CALIS to be an ideal measure for use within the standard set of measures for the assessment of anxiety and depression in children and young people (4–24 years). However, the working group highlighted the limitation of the measure’s focus on anxiety. In addition to the high comorbidity between clinical depressive and anxiety disorders, it is common for sub-clinical levels of depressive symptoms to also be reported alongside anxiety symptoms [25, 26]. Anxious children with comorbid depression are more likely to report more severe impairment and show more severe anxiety and specific depressive symptoms (e.g., negative mood, anhedonia) than anxious children without comorbid depression [14, 27]. Diagnostically, there is also significant overlap between the symptoms of anxiety and depressive disorders, such as negative affect [28] and the rumination of negative thoughts [29]. This overlap can make it difficult for children to differentiate between anxiety and depressive symptoms, as well as to describe their differential impact. For some children, these symptoms are experienced holistically, and the interference they cause is often analogous. Due to the significant impairment resulting from comorbid anxiety and depressive disorders, it is important to be able to measure interference from both anxiety and depressive disorders in children. There is currently no measure that adequately assesses interference from anxiety and or depression symptoms in children and adolescents.

In direct response to the measurement gap identified by the ICHOM working group, we sought to develop a new brief measure of life interference of anxiety and depressive symptoms. Adapting the widely used CALIS measure, we developed the Child Anxiety and Depression Life Interference Scale (CADLIS) to assess life interference of depressive and anxiety disorders in children and adolescents. Based on previous validation studies of the CALIS, it was hypothesised that CADLIS would demonstrate good psychometric properties through adequate internal consistency, convergent and divergent validity, and display a two-factor model of in the home interference and outside the home interference for both the parent and child measure.

## Method

### Participants

A total number of 1299 participants (672 parents and 627 children) participated in the study. Data on 1130 children were analysed: 503 from parent-only reports, 458 from child-only reports and 169 from matched parent and child reports. Across the total sample, children (*n* = 1130) were aged between 4 and 18 and had a mean age of 12.46 years (*SD* = 3.63), and there was an even split of secondary school (50.5%) and primary school students (49.5%). Participants were from across Australia and a small number of participants were international. Just under half of the sample resided in NSW (47%) including regional and rural areas, with 24% from Victoria, 13% from Queensland and a small fraction (0.6%) of participants reported living outside of Australia. 78% of parents reported that their family were self-isolating to some extent due to the COVID19 pandemic. Descriptive statistics for the total sample are presented in Table 1.

**Table 1:** Demographic characteristics of children and parents

Variable	Child	Parent
Gender, *n* (%) Male Female Non-Binary Other	*n* = 1107* 619 (55.93) 476 (42.99) 7 (0.63) 5 (0.45)	*n* = 660 193 (26.47) 483 (73.18) 2 (0.29)
Ethnicity, *n* (%)		*n* = 650
Oceanic North-West European Southern and Eastern European North African and Middle Eastern		403 (61.94) 89 (13.71) 39 (6.01) 11 (1.69)
South-East Asian North-East Asian Southern and Central Asian People of the Americas Sub-Saharan African		37 (5.70) 26 (4.01) 32 (4.93) 7 (1.08) 6 (0.92)
Aboriginal and/or Torres Strait Islander, *n* (%)	*n* = 1108	*n* = 650
Yes No	59 (5.42) 1049 (94.58)	29 (4.45) 621 (95.55)
Language spoken at home, *n* (%)	*n* = 636	*n* = 649
English Italian Greek Cantonese Arabic Mandarin Vietnamese Other	595 (93.55) 2 (0.31) 2 (0.31) 8 (1.26) 1 (0.16) 1 (0.16) 7 (1.10) 20 (3.15)	598 (92.14) 1 (0.15) 1 (0.15) 8 (1.23) 3 (0.46) 2 (0.31) 8 (1.23) 28 (4.31)
Gross weekly income, *n* (%)		*n* = 649
$0–$999 $1000-$2399 $2,400–$3,999 $4,000–$6,399 $6,400–$8000+		110 (17.10) 279 (42.90) 152 (23.42) 57 (8.78) 51 (7.86)
IRSD**, *n* (%)	*n* = 514	
1–3 4–7 8–10	79 (15.36) 105 (38.73) 230 (46.28)	

For parent–child dyads, parent reports on demographics were used for the demographic report. The differing n’s represent missing data where participants chose not to disclose some demographic information. *Sample is made up of 494 from parent only reports, 445 from child only reports and 169 from matched parent and child reports (*n* = 1108). **IRSD = The Index of Relative Socio-economic Disadvantage, where lower scores represent higher disadvantage (Australian Bureau of Statistics [30]). Ethnicity and weekly income were only collected from parent samples, and IRSD was only collected from child reports

To achieve a large and diverse sample, participants were recruited from four channels: schools (*n* = 320), clinic (*n* = 41), panel (*n* = 483) and community (*n* = 290).

### School sample

Children and their parents were recruited from independent primary and high schools from the Greater Sydney area. Eight schools were invited to participate via email, two consented to participate. Parents were sent information about the study and an opt-out consent form; with a two-week turnaround. Students completed the survey online during class under the supervision of a teacher and the researchers during Term 3 2020. Each student provided informed consent. Parents were sent the online survey link via the school to complete in their own time.

### Community sample

The community sample was obtained through advertisements on social media. Parents provided consent for themselves and their child. Children 14 and above could provide their own consent without parental consent to participate.

### Panel sample

The panel sample was obtained through a panel provider CINT.com. The survey link was distributed through the platform, and parents provided consent for themselves and their child to participate.

### Clinic sample

The clinical sample was obtained through families seeking treatment for anxiety at the Centre for Emotional Health, Macquarie University. Clinicians provided information on the study to the family during the assessment. If consent was provided, the survey was then included into their assessment package. Children were assisted by the researcher to complete the survey. The family’s clinic ID was used to match parent–child responses.

## Measures

### Child anxiety and depression life interference scale (CADLIS)

The CADLIS is an adapted measure of the CALIS [13]. The CADLIS assesses the interference from anxiety and depressive disorders in a child's life and their parent’s life and includes two versions for primary school children aged 7–12 (CADLIS-C- PS) and secondary school students aged 12–18 (CADLIS-C-SS) and a parent measure for children aged 4–18 (CADLIS-P). It is brief and designed to be used independently or to be used in conjunction with other symptom measures. Through consultations with the International Consortium for Health Outcomes Measurement and the authors of the CALIS, additional changes were made to the measure to include items specific to depressive disorders such as an anhedonia and sleep item, so interference from depressive disorders was adequately captured. (Note: the distress item was removed to enable the measure to be specific to life interference). Further changes were made to make the scale applicable across cultures by rewording three items, the first to include extended family, the second to rephrase “mates” to “friends” and lastly to reword “recess and lunch” to “at school”.

A pilot study was conducted with 18 children aged 7–17 recruited via a convenience sample to examine the face validity of using the statement ‘feeling anxious and depressed’ accompanied by an image that contained a textbox with the feelings associated with anxiety and depression. The results of the pilot study revealed that the statement ‘feeling anxious and depressed’ accompanied by the age-appropriate picture description was adequately comprehended by all ages surveyed.

The CADLIS-C includes nine items that examine interference related to their family, social and academic functioning, e.g. “make it difficult for you to get your schoolwork done”. Children rated the extent to which each item was true for them on a five-point scale (*Not at all, only a little, some, quite a lot, a great deal*). The CADLIS-P includes 16 items related to interference in the child’s life and parent’s life, using the stem question "How much does anxiety and depression interfere with your child's everyday life in the following areas?" and "How much does your child's anxiety and depression interfere with your everyday life in the following areas", followed by sub-questions that related to family, social and occupational functioning. Parents rated the extent to which each item was true for them and their child on the same five-point scale. A total score is calculated by summing the items, with higher scores indicating higher levels of interference. Child scores range from 0–36 and parent scores range from 0- 64.

### Revised child anxiety and depression scale (RCADS-25)

The RCADS-25 [31] measures anxiety and depressive symptoms in children. Both the parent and child report have 25 items, with two subscales measuring anxiety and depression, respectively. The RCADS-25 assesses the frequency of symptoms, with children and parents responding to items on a four-point Likert Scale the frequency of symptoms for them/their child (*Never, sometimes, often, always*). The total score is calculated by summing the 25 items and then converting the raw score into a t-score standardised for gender and grade, with higher scores indicating a higher frequency of symptoms. For this study, high RCADS-25 scores were characterised by scores of 65 and above [32]. The measure has proven to correlate with DSM diagnoses of anxiety and depression and demonstrate high reliability in samples of children and adolescents across clinical and community samples [33]. Cronbach’s alpha for this study was high for both child and parent reports (α C = 0.95, α P = 0.93).

### Sheehan disability scale (CSDS/-P)

The CSDS/-P [8] is a measure which assesses impairment in three domains: work/school, family and social functioning in addition to the parents functioning. It is an adaptation of the original SDS [34]. The parent report has five items, and the child report has three items. Both children and parents rate the extent to which each item is true for them/their child on an eleven-point scale of 0 (*Not at all*) to 10 (*Extremely*). The total score is calculated by summing the item ratings with higher scores associated with more significant impairment. The CSDS/-P has shown strong construct validity in measuring interference from internalising disorders as it is significantly and positively related to anxiety symptoms, and not significantly related to externalising disorders [8]. The CSDS/-P demonstrated high internal consistency for both parent and child reports (P α = 0.95, C α = 0.90) consistent with prior studies using the measure [8]. Child scores range from 0 to 30, parents’ scores range from 0 to 50.

### Strengths and difficulties questionnaire (SDQ-P/C)

The SDQ-P/C [35] measures psychopathology and prosocial behaviour in children through five dimensions; emotional symptoms, hyperactivity-inattention, conduct problems, peer problems and prosocial behaviour. It is a brief 25-item measure in which parents and children rate on a three-point scale the extent to which items are true for them/their child (*Not true, Somewhat true, Certainly True*). For this study, the externalising subscale (10 items) and emotional subscale (five items) were used. The externalising subscale is calculated by summing the hyperactivity and conduct subscale together and is recommended for use in community samples [36]. Higher scores are associated with higher emotional and conduct problems respectively. It has moderate reliability and discriminant validity, thus can be used for screening for internalising and externalising symptoms [35]. For this study the emotional and externalising subscales demonstrated high reliability (α CEm = 0.85 CExtern = 0.75, α PEm = 0.85, PExtern = 0.82).

### Demographics and attention checks

Child participants reported demographic information, including their grade, age, gender and ethnicity. Parent participants reported the same demographic information for their child and their own age, gender, ethnicity, income, postcode and isolation status, as the study was conducted during the COVID-19 pandemic. Embedded in the questionnaire was an attention check which asked participants to select ‘sometimes’.

## Procedure

Ethics approval was granted by the Macquarie University Human Research Ethics Committee. Each sample population had a separate recruitment and consent procedure. The inclusion criteria for the study were based on parental status and child age: Parents who had a child aged 4–18 were invited to participate as were children aged 7–18. Although the study was advertised to families living in Australia, location did not serve as an exclusion criterion. The survey was conducted online via LimeSurvey. All participant information was de-identified, a secret code was created for each child to link with their parent’s report. Upon completion of the survey, parents and children were provided with a debrief statement that provided mental health resources. Participants from the school, community and clinic samples had the opportunity to enter a draw to win an iPad whereas the panel sample was provided monetary reimbursement via the panel provider. All participants were free to withdraw at any time without penalty.

### Data analysis strategy

A confirmatory factor analysis (CFA) was applied to support the underlying factor structure of the CADLIS based on previous results from the CALIS. The CFA was conducted with maximum likelihood estimation, and the participant-to-parameter ratio of 25.08 indicated adequate sample size [37]. A two-factor structure was predicted (inside and outside the home) based on prior factor structure of the CALIS [13]. Model fit was assessed with multiple indices, with the following indicating acceptable fit: comparative fit index (CFI) > 0.90 [38], Tucker-Lewis index (TLI) > 0.95 (Bentler [39]), Standardized Root Mean Square Residual (SRMR) < 0.08, root mean square error of approximation (RMSEA) < 0.08 [40] and a ratio of five or less for the χ2 to the degrees of freedom [41]. As the original model displayed poor model fit, modification indices were examined and implemented incrementally until model fit was achieved. Modification indices were added if they had a large, expected model change corresponding to a significant improvement (*p* < 0.001) in chi-square and theoretical support.

A CFA was applied to the parent version (CADLIS-P), although for this measure a three-factor solution was tested (inside the home, outside the home, and parent life) based on prior results from the CALIS [13]. Due to poor model fit for the predicted three-factor model, and no modification indices which significantly improved the model fit, an exploratory factor analysis (EFA) was conducted to examine an alternate possible structure. Principal Axis Factoring (PAF) with oblimin rotation was used due to the high probability of correlations between the factors [42]. Finally, internal consistency and agreement between parent and child reports were evaluated, along with convergent and divergent validity using Pearson’s correlation and Fisher’s Z-transformation to examine differences in the correlation between the CADLIS and measures of internalizing symptoms and externalising symptoms. We expected the CADLIS to be more strongly related to measures of internalising symptoms (RCADS, SDQ-E and CSDS) than externalising symptom measures (SDQ-Ext). Correlations were run separately between parent-reported and child-reported measures. Similarly, given the violation of the assumption of normality, a Mann–Whitney U was conducted to examine differences in CADLIS scores between those who scored high or low on the RCADS-25 to determine if the CADLIS could differentiate between clinical and non-clinical impairment to assess the concurrent validity of the CADLIS. All analyses were performed using Stata/IC 16.1.

## Results

### Data management

The four samples were merged into one data set. As the data set was utilised for other studies, only participants who completed the CADLIS were included in the sample, parent and child dyads were matched via the secret code. The original data set contained 1543 participants. A total of 244 participants were removed due to failing item attention checks to ensure data quality. Missing data at the item level was dealt with by prorating total scores using the mean when at least 80% of the measure of interest was completed. Participants who completed less than 80% of a measure were dropped at a measure level but not from the data set (*n* = 94). Thus, each measure has a different total sample. The final data set contained 1299 participants (parent *n* = 672, child *n* = 627).

### Descriptive statistics

As expected, there were significant differences between the demographics and measure scores across the different samples. These are provided in the supplementary materials (Supplement A). We have provided the means and standard deviations for each measure in Table 4.

### CADLIS-C factor structure

The hypothesised two-factor model did not adequately fit the data (RMSEA = 0.239; CFI = 0.780, TLI = 0.707, SRMR = 0.414, χ^2^ = 9991.999, *df* = 27, *p* < 0.001). As model fit was poor, model fit indices were examined, and the addition of the three covariances was suggested. The first covariance (modification index = 424.752) was between the two latent factors (inside the home) and (outside of the home). Given the high modification indices, and resultant high inter-factor correlation (0.95) suggesting little differentiation between the two-factors, a one-factor model was also tested to determine if a single “life interference factor” was a better representation of the model. The one-factor model where all items loaded onto a single factor of life interference displayed slightly better model fit than the two-factor model (RMSEA = 0.128; CFI = 0.937, TLI = 0.916, SRMR = 0.041, χ^2^ = 303.527, *df* = 27, χ2/df = 11.2, *p* < 0.001), however, it was still not within the required range for model fit, thus modification indices were explored. A bifactor model and higher-order model were also attempted, however, these models did not converge. The first covariance (modification index = 120.631) was added to the model between the residuals of item one (“How much does feeling anxious and/or depressed make it difficult for you to get along with your parents?”) and item two (“How much does feeling anxious and/or depressed make it difficult for you to get along with other family members?”) displayed better fit however, the RMSEA was still not within optimal range (RMSEA = 0.097; CFI = 0.965, TLI = 0.952, SRMR = 0.031, χ2 = 179.313, df = 26, χ2/df = 6.90, p < 0.001). The second covariance (modification index = 27.189) was added to the model between the residuals of item three (“to be with friends outside of school?”) and five (“to be with other children at school?”) however the RMSEA was still not within range (RMSEA = 0.090; CFI = 0.971, TLI = 0.959, SRMR = 0.029, χ2 = 153.069, df = 25, χ2/df = 6, p < 0.001). A final covariance (modification index = 37.175) was then added between the residuals of item four (“to get your schoolwork done”) and item nine (“to get to sleep, stay asleep or wake up on time”). The final model was re-run with three covariances. The one-factor model with three covariances fit adequately (RMSEA = 0.078; CFI = 0.979, TLI = 0.969, SRMR = 0.025, χ2 = 115.832, df = 25, χ2/df = 4.83, p < 0.001). All items loaded significantly (*p* < 0.05) and strongly within the model (λs = 0.68–0.86), factor loadings are displayed in Table 2. The relationship between the residuals of item two and three was significant and moderate (0.44), and the relationship between the residuals of three and five, and four and nine was significant and weak (0.22, 0.26). The one-factor model with three additional covariances was retained as the optimal model for the CADLIS-C, the final model is displayed in the supplementary material (Supplement B).

**Table 2:** Standardised factor loadings for the CADLIS-C

Factor	Item	Factor loading
Interference	1. Get along with your parents	.74
	2. Get along with other family (e.g. your brothers, sisters or grandparents)	.73
	3. Be with friends outside of school	.84
	4. Get your schoolwork done	.80
	5. Be with other children at school	.86
	6. Take part in activities such as sport, dance, music or art	.83
	7. Make it difficult for you to do fun things like going to parties, movies or holidays?	.83
	8. Complete daily activities such as getting ready for school, getting chores done and homework	.84
	9. Get to sleep, stay asleep or wake up on time	.68

The stem question for questions 2–10 was: “How much does feeling anxious or depressed make it difficult for you to do the following?” Standardised loadings are presented

### CADLIS-P factor structure

The hypothesised three-factor model for the parent CADLIS report did not adequately fit the data in the CFA analysis (RMSEA = 0.172; CFI = 0.767, TLI = 0.734, SRMR = 0.448, χ^2^ = 2482.790, *df* = 119, *p* < 0.001). There were no modification indices that could be made to improve model fit significantly; thus it was decided to run an exploratory factor analysis to examine the factor structure of the model. Principal Axis Factoring (PAF) was conducted on the 16 items with oblimin rotation. The PAF proposed a two-factor model from the examination of eigenvalues and the screeplot. Horn’s analysis proposed a one-factor model with only one adjusted eigenvalue above one, the second eigenvalue, however, was 0.98 thus the two-factor model was selected. The two-factor model accounted for 69% of variance. The Kaiser–Meyer–Olkin measure verified sampling adequacy, total KMO = 0.95, and individual items had KMO values above 0.93, which was within the excellent range [43]. Bartlett’s test of sphericity, which tests the overall significance of all the correlations within the correlation matrix, was significant (χ 2 (120) = 9708.832 p < 0.001), indicating that it was appropriate to use the factor analytic model on this set of data. The determinant was above < 0.00001 indicating there is no significant multicollinearity present.

Examination of the two-factor model (CADLIS-P) showed that after oblimin rotation, items regarding child life interference loaded onto factor one, and items regarding parent life interference loaded onto factor two. Item 10 ‘sleep’ loaded onto the parent life interference, for the factor loadings it was retained within the parent factor, however, for scoring purposes it was allocated to child life as there is an equivalent item on the child measure. The factor loadings and eigenvalues are reported in Table 3.

**Table 3:** Factor loadings of the exploratory factor analysis with oblimin rotation and eigenvalues for the CADLIS-P

Item	Factor Loading	
	1	2
Factor 1: Child Life Interference		
1. Getting on with parents	**0.42**	0.40
2. Getting along with other family (e.g. your brothers, sisters or grandparents)	**0.47**	0.32
3. Being with friends outside of school	**0.78**	0.05
4. Performance in the classroom	**0.90**	0.08
5. Interacting with peers at recess and lunch	**0.89**	0.01
6. Taking part in activities such as sport, dance, music, art	**0.86**	0.03
7. Doing enjoyable activities like going to parties, movies or holidays	**0.78**	0.04
8. Daily activities such as getting ready for school, getting chores done and homework	**0.53**	0.30
Factor 2: Parent life interference		
9. Sleep (e.g. getting to sleep or waking up on time)	0.36	**0.42**
10. Your relationship with your partner or a potential partner	0.06	**0.88**
11. Your relationship with extended family	0.07	**0.91**
12. Your relationship with friends	0.00	**0.85**
13. Your career (choice to work, how many hours you do or how often you miss work)	0.06	**0.84**
14. Your ability to go out to activities/events with your child	0.17	**0.70**
15. Your level of stress	0.14	**0.69**
16. Your confidence as a parent	0.16	**0.65**
*Eigenvalues*	10.73	1.12

*N* = 650, Extraction method: PAF, 2 factors extracted, 10 iterations. Child life interference questions were started with the stem question: How much does anxiety or depression interfere with your child’s everyday life in the following areas? Parent life questions were started with the stem question: How much does your child's anxiety or depression interfere with your everyday life in the following areas? Factor on which item loads is indicated in bold

### Internal consistency and inter-rater reliability

McDonald's omega for the CADLIS-C was 0.94. McDonald’s omega was 0.96 for the total CADLIS-P report, 0.94 for the child life interference subscale and 0.94 for the parent life interference subscale. Agreement between parent and child CADLIS reports was examined using the average intra-class correlations (ICC) for the CADLIS total report between 169 matched parent–child dyads. The ICC for parent–child agreement and descriptive statistics for the measures of interest are reported in Table 4. Across the parent reports, 86.49% of children with elevated depression, and 85.76% with elevated anxiety symptoms, were also identified as having elevated comorbid anxiety and depression symptoms. Similarly, across child reports 81.65% and 79.82% of the high anxiety and high depression symptom groups respectively were in the high total RCADS25 group.

**Table 4:** Sample means for symptom and interference measures

Measure	Child report			Parent report				ICC
	*n*	*M*	*SD*	*n*	*M*	*SD*	*n*	
RCADS25	627			672				
Total		20.00	14.35		14.44	11.41		
Anxiety		11.44	8.46		8.53	6.87		
Depression		8.55	6.51		5.91	5.22		
CSDS	590	8.63	8.22	640	12.49	13.44		
SDQ-Emotional	589	3.60	2.98	636	3.10	2.90		
CADLIS	627			672				
Total		11.78	9.86		16.44	15.10	169	.78**
Child Life					9.91	9.04		
Parent Life					6.52	6.81		

RCADS25 = Revised Child Anxiety and Depression Scale, CSDS = Child Sheehan Disability Scale, SDQ-E = Strengths and Difficulties Questionnaire– Emotional subscale, CADLIS = Child Anxiety and Depression Life Interference Scale. ICC = average intraclass correlation

**p <.001

### Convergent and divergent validity

The CADLIS-P correlated positively and strongly with the RCADS25-P, SDQ-P emotional subscale and the CSDS-P. The CADLIS-C correlated positively and moderately with the SDQ-C emotional subscale and CSDS and strongly with the RCADS25-C. The correlation coefficients and their significance levels are reported in Table 5.

**Table 5:** Correlations between the child anxiety and depression life interference scale and other measures of interference and symptoms

	Measures of internalising symptoms/interference			
	RCADS25 SDQ-E CSDS			SDQ-Ext
*CADLIS-C*	*n* = 627	*n* = 589	*n* = 590	*n* = *566*
Total score	.67**	.60**	.65**	.43**
*CADLIS-P*	*n* = 672	*n* = 636	*n* = 640	*n* = *598*
Total score	.77**	.74**	.77**	.55**
Parent Life	.70**	.66**	.74**	.53**
Child Life	.73**	.70**	.74**	.53**

Coefficients correlate same reporter for each pair. RCADS25 = Revised Child Anxiety and Depression Scale, CSDS = Child Sheehan Disability Scale, SDQ-E = Strengths and Difficulties Questionnaire– Emotional subscale, CADLIS = Child Anxiety and Depression Life Interference Scale, SDQ-Extern = Strengths and Difficulties Questionnaire– Externalising subscale

**p <.001

Divergent validity was examined using Fisher’s z-transformation. For the parent report, the CADLIS correlated significantly more strongly with the RCADS-25 than the SDQ-Externalising subscale, *z* = 8.106, *p* < 0.01 (one-tailed). Similarly, for the child report there was a significant difference in the correlations between the CADLIS and RCADS-25 and the CADLIS and the SDQ-Externalising subscale in the same direction *z* = 7.816, *p* < 0.01 (one-tailed). The correlation between internalising and externalising measures was compared between the CADLIS and CSDS using Fisher’s Z-transformation to test for incremental validity. For the parent report, the SDQ-Externalising report correlated significantly less with the CADLIS than the CSDS, *z* = −1.82 *p* = 0.04 (one-tailed). The RCADS-25 correlated significantly more strongly with the CADLIS compared to the CSDS,* z* = 2.51, *p* = 0.01 (one-tailed). For the child report, the RCADS-25 correlated significantly more strongly with the CSDS than the CADLIS, *z* = 0.97, *p* = 0.17 (one-tailed). There was a significant difference in the correlations between the SDQ-Externalising subscale, CADLIS and CSDS. The SDQ-Externalising subscale correlated significantly less with the CADLIS compared to the CSDS *z* = −2.90, *p* < 0.01 (one-tailed).

### Differentiation between high and low RCADS25 scores

An independent samples t-test was planned to compare the CADLIS total mean scores between the RCADS-25 high symptom (RCADS-25-C $\ge$ 65) and low symptom group (RCADS-25-C < 65). Normality of the RCADS25-P/C and the CADLIS-P/C were first examined using the Shapiro–Wilk test and the data were found to be non-normal (*p* < 0.05), therefore a non-parametric test was used. Mann–Whitney U was deemed the most appropriate analysis. Parents who rated their child in the high RCADS-25 symptom group had higher CADLIS-P scores (*Mdn* = 33) than the low symptom RCADS-25 group (*Mdn* = 9). A Mann Whitney test indicated that this difference was statistically significant, *U* (*N*_high RCADS25_ = 162, *N*_low RCADS25_ = 510) = 4,621,633.81, *z* = −16.15, *p* < 0.001. Children who self-reported high RCADS-25 scores reported higher CADLIS-C scores (*Mdn* = 20) than the low symptom RCADS-25 group (*Mdn* = 6). A Mann Whitney test indicated that this difference was statistically significant, *U* (*N*_high RCADS25_ = 106, *N*_low RCADS25_ = 503) = 26,666,128.85, *z* = −12.72, *p* < 0.001.

## Discussion

The present study indicates support for the validity, and reliability of the CADLIS-C/P an adapted measure of the CALIS, developed to assess the life interference caused by anxiety and depression in children and adolescents. Further research is required to confirm the factor structure of the CADLIS. The confirmatory factor analysis of the CADLIS-C did not support the two proposed sub-scales of life interference within the home and outside of the home, rather a one-factor model of “life interference” was found to be the most appropriate model. Previous studies of the CALIS have found high inter-factor correlations suggesting commonality between the subscales [13, 44, 45]. Thus, a one-factor model is not surprising. The factor structure may be accounted for due to the impact of COVID-19 restrictions which were in place during data collection [2, 3, 46]. Within our sample, 78% of people were self-isolating to some extent. Thus, the distinction between inside the home and outside of the home during data collection was less well defined for both children and parents, compared to when the factor structure of the CALIS was examined in 2013, which could explain the factor structure.

The hypothesised CADLIS-P three-factor model, differentiating life interference inside the home, outside the home and parent life interference factors, did not adequately fit the data. The EFA provided mixed results for the factor structure with recommendations of one (Horn’s analysis) or two factors (eigenvalues and screeplot). Given the theoretical support for the two-factor model with the CALIS factors differentiating between parent and child interference a two-factor model of child and parent life interference was selected and provided a better fit than the three-factor solution. Although contrary to previous CALIS research [13, 47], it is consistent with findings from the CSDS [8] and the singular factor structure for the CADLIS-C, indicating that interference in a child’s life can be measured holistically.

The CADLIS-C/P demonstrated significant moderate to strong convergent validity in its relationships with other symptom and interference measures and significantly lower correlations between the CADLIS and measures of externalising symptoms. Divergent validity was demonstrated with significantly lower correlations between the CADLIS and externalising symptoms compared to the correlation between the CADLIS and internalising measures. The moderate correlation between the CADLIS-P and the SDQ-Externalising subscale is to be expected as there are also high levels of *heterotypic comorbidity* between internalising and externalising disorders and is in line with other interference measures [8, 48]. Correlations with externalising symptoms were lower than with internalising symptoms, supporting the divergent validity of the CADLIS.

The CADLIS demonstrated incremental validity when compared to the CSDS for divergent and convergent validity for the parent report and divergent validity for the child report. Indicating the CADLIS provides unique information regarding interference from anxiety and depressive disorders when compared to a global impairment measure. Further, children with higher anxiety and depression symptoms reported higher levels of interference, indicating that the CADLIS can differentiate between clinically significant and non-significant interference. There was a high level of comorbidity present within the sample with between 79 and 86% of the sample meeting criteria for threshold or clinical symptoms of both anxiety and depression. This further justifies the need for a measure of impairment for both anxiety and depression in children.

The CADLIS displayed excellent internal reliability within the subscales and total scale [49], and good inter-rater reliability, with strong positive correlations between child and parent reports. The moderate to strong relationship between parent–child responses is consistent with other studies which have found that observable behaviour such as interference leads to stronger relationships between parent–child responses [19].

### Limitations

The current study had a small clinical sample and may not be representative of families seeking treatment. The over-representation of high-income families limits the generalisability of the study to broader community settings. A subset of the sample was from a panel provider CINT.com (*n* = 483), and there was no way to ensure that a child completed the survey. However, 309 children completed the measure under supervision.

The use of modification indices to improve model fit is another limitation. Modification indices can lead to model overfitting and may increase the risk of capitalizing on chance correlations, resulting in less generalisable and replicable factor solutions. The post hoc analyses allowed for a better model fit to be determined and all model covariances had a theoretical underpinning, refer to Supplement C for further information on theoretical justifications. Future research is required to confirm the factor structure.

Finally, the CADLIS provides an assessment of the total impact of anxiety and depressive symptoms, rather than the impact specifically attributed to either depression or anxiety alone.

## Conclusion

The CADLIS is a reliable measure that demonstrates evidence of convergent validity for assessing life interference from anxiety and depressive symptoms in children aged 4–18.

The CALDIS is a short measure with only 9 items for children and 16 for parents thus it is a low survey burden and can easily be paired with a symptom measure or used independently without the need for a measure of symptoms. Importantly, the measure provides unique information regarding life interference when compared to a global impairment measure and can differentiate children with high and low anxiety and depressive symptoms. The factor structure indicated a total score for life interference is warranted for child report (CADLIS-C). For the parent report (CADLIS-P), the total score can be calculated in addition to a score for parent life interference and child life interference. With further testing in clinical samples, the CADLIS has the potential to be used during diagnostic assessments and as a screener tool.

## Data Availability

No datasets were generated or analysed during the current study.
